# Metabolic engineering *Escherichia coli* for efficient production of icariside D2

**DOI:** 10.1186/s13068-019-1601-x

**Published:** 2019-11-06

**Authors:** Xue Liu, Lingling Li, Jincong Liu, Jianjun Qiao, Guang-Rong Zhao

**Affiliations:** 1 Frontier Science Center for Synthetic Biology and Key Laboratory of Systems Bioengineering (Ministry of Education), School of Chemical Engineering and Technology, Tianjin University, Yaguan Road 135, Jinnan District, Tianjin, 300350 China; 2 SynBio Research Platform, Collaborative Innovation Centre of Chemical Science and Engineering (Tianjin), Tianjin University, Yaguan Road 135, Jinnan District, Tianjin, 300350 China

**Keywords:** Icariside D2, *Escherichia coli*, Coculture, Synthetic biology, Metabolic engineering

## Abstract

**Background:**

Icariside D2 is a plant-derived natural glycoside with pharmacological activities of inhibiting angiotensin-converting enzyme and killing leukemia cancer cells. Production of icariside D2 by plant extraction and chemical synthesis is inefficient and environmentally unfriendly. Microbial cell factory offers an attractive route for economical production of icariside D2 from renewable and sustainable bioresources.

**Results:**

We metabolically constructed the biosynthetic pathway of icariside D2 in engineered *Escherichia coli*. We screened the uridine diphosphate glycosyltransferases (UGTs) and obtained an active RrUGT3 that regio-specifically glycosylated tyrosol at phenolic position to exclusively synthesize icariside D2. We put heterologous genes in *E. coli* cell for the de novo biosynthesis of icariside D2. By fine-tuning promoter and copy number as well as balancing gene expression pattern to decrease metabolic burden, the BMD10 monoculture was constructed. Parallelly, for balancing pathway strength, we established the BMT23–BMD12 coculture by distributing the icariside D2 biosynthetic genes to two *E. coli* strains BMT23 and BMD12, responsible for biosynthesis of tyrosol from preferential xylose and icariside D2 from glucose, respectively. Under the optimal conditions in fed-batch shake-flask fermentation, the BMD10 monoculture produced 3.80 g/L of icariside D2 using glucose as sole carbon source, and the BMT23–BMD12 coculture produced 2.92 g/L of icariside D2 using glucose–xylose mixture.

**Conclusions:**

We for the first time reported the engineered *E. coli* for the de novo efficient production of icariside D2 with gram titer. It would be potent and sustainable approach for microbial production of icariside D2 from renewable carbon sources. *E. coli*–*E. coli* coculture approach is not limited to glycoside production, but could also be applied to other bioproducts.

## Background

Icariside D2 (4-*O*-β-d-glucoside of tyrosol), an active natural product, was first isolated from traditional medicinal herb *Epimedium diphyllum* [1]. *Epimedium* plants have been widely used for treatment of osteoporosis, nervous dysfunction, hypertension, and cardiovascular and inflammatory diseases [2, 3]. Recently, more attentions have been paid on the pharmacological studies of icariside D2. It has been reported that icariside D2 had synergistic inhibitive effect on angiotensin-converting enzyme [4] and remarkable anticancer activity to kill leukemia cells in vitro [5], demonstrating the therapeutic and nutraceutical potential of icariside D2 in the health care industry.

Although icariside D2 has been identified from several plants such as *Epimedium* [1], *Apium graveolens* [4], *Annona glabra* [5], *Ficus microcarpa* [6], *Tinospora sinensis* [7], and *Rhodiola crenulata* [8], extraction of icariside D2 suffers low yields for the low content and limited plant resources. Chemical synthesis for glycoside products has always been frustrated by the diversity of stereochemistries and regiochemistries, and generally requires multiple protection and deprotection steps to achieve regio-selectivity in glycosylation [9]. Enzymatic synthesis of natural glycosides is limited by the prohibitive nucleotide-phosphate glucose and thus is currently impractical to be used at a larger scale [10]. Recently, the strategy of synthetic biology has been applied to metabolically engineer microbes as cell factories for de novo biosynthesis of glycosides. Salidroside [11–13], anthocyanin [14], astragalin [15], and flavonoid rhamnosides [16] were biosynthesized in engineered *Escherichia coli*.

**Fig. 1:**
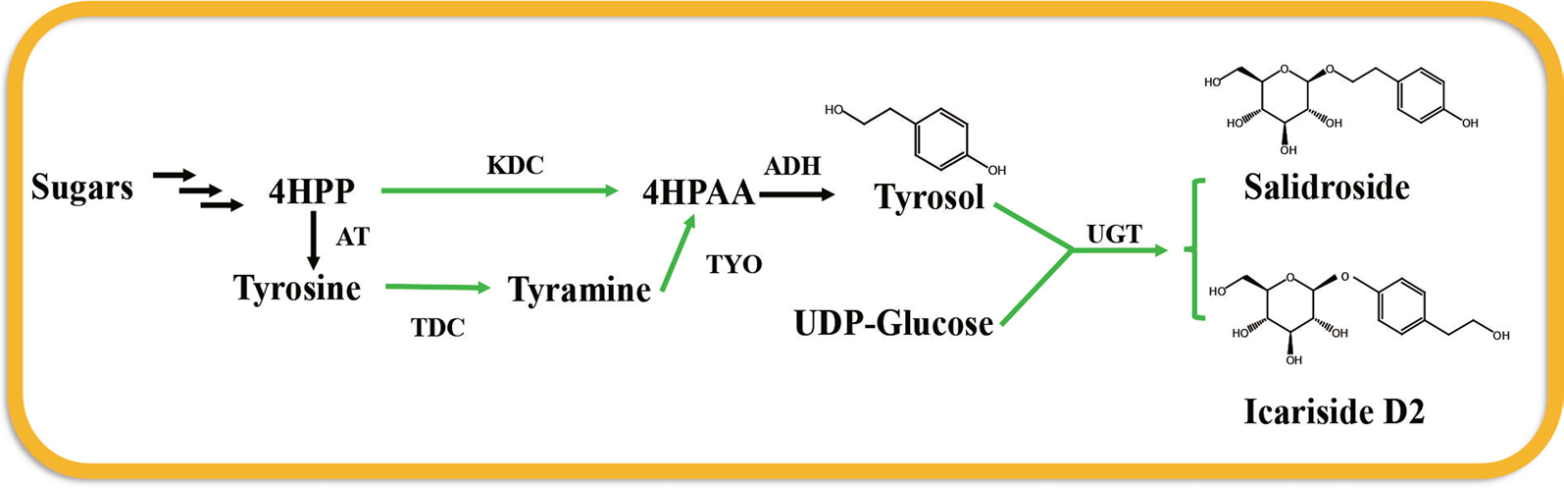
The artificial biosynthetic pathway of tyrosol glucosides in *E. coli*. Green arrows indicated the reactions catalyzed by the heterologous enzymes. *4HPP* 4-hydroxyphenylpyruvate, *4HPAA* 4-hydroxyphenylacetaldehyde, *4HPA* 4-hydroxyphenylacetate, *UDP glucose* uridine 5, 9-diphosphoglucose. *AT* aminotransferase, *KDC* keto acid decarboxylase, *TDC* tyrosine decarboxylase, *TYO* tyramine oxidase, *ADH* alcohol dehydrogenase

Glycosylation of tyrosol on phenolic or alcoholic positions by regio-selective UGTs would result in icariside D2 or salidroside, which are structural isomers (Fig. 1). Two de novo biosynthetic pathways of tyrosol have been developed via 4-hydroxyphenylacetaldehyde (4HPAA) which is reduced to tyrosol by the native alcohol dehydrogenase (ADH) in *E. coli* and the endogenous expression was sufficient for tyrosol production [13]. 4HPAA would be synthesized from 4-hydroxyphenylpyruvate (4HPP) catalyzed by keto acid decarboxylase (KDC) [17], or from tyrosine by sequential reactions catalyzed by tyrosine decarboxylase (TDC) and tyramine oxidase (TYO) [18]. Between them, the KDC has been demonstrated the more efficient one in *E. coli* [19]. In our previous study, the regio-glycosylation of tyrosol on alcoholic position catalyzed by AtUGT85A1 together with the KDC resulted in high production of salidroside in engineered *E. coli* [13]. Here, we for the first time reported the engineered *E. coli* for the efficient production of icariside D2. A regio-specific glycosyltransferase toward phenolic position of tyrosol was obtained and used for the construction of icariside D2 biosynthetic pathway. Metabolically engineered *E. coli* monoculture produced 3.80 g/L of icariside D2 using glucose as sole carbon source, and engineered *E. coli*–*E. coli* coculture produced 2.92 g/L of icariside D2 using glucose–xylose mixture as carbon source in fed-batch shake-flask fermentation.

## Results and discussion

### Glycosyltransferase screening for regio-selective biosynthesis of icariside D2 from tyrosol

Icariside D2 could be synthesized by the regio-selective UGT through glycosylation reaction on phenolic position of tyrosol aglycone with the UDP glucose as glycosyl donor. Several UGTs from plants and bacteria have been identified and demonstrated for the capacity of glycosylating tyrosol with the regio-promiscuity. For example, RsUGT73B6 [11] and RrUGT17 [20] from *Rhodiola*, and YjiC [13] from *Bacillus licheniformis* glycosylated tyrosol on both phenolic and alcoholic positions to simultaneously produce icariside D2 and salidroside, and YjiC was most effective among them (Additional file 1: Fig. S1). Considering the relative high content of tyrosol glycosides and rich UGT resources in *Rhodiola* genus [21], we expected to obtain a regio-specific UGT from *Rhodiola* plants to efficiently catalyze tyrosol to icariside D2 in *E. coli*. Accordingly, RcUGT1 from *Rhodiola crenulata* [8] and RrUGT3 from *Rhodiola rosea* [20] were chosen. Additionally, RsUGT72B14 from *Rhodiola sachalinensis* that had high in vitro enzymatic activities to glycosylate tyrosol [22] was also investigated for the potential role in icariside D2 biosynthesis.

**Fig. 2:**
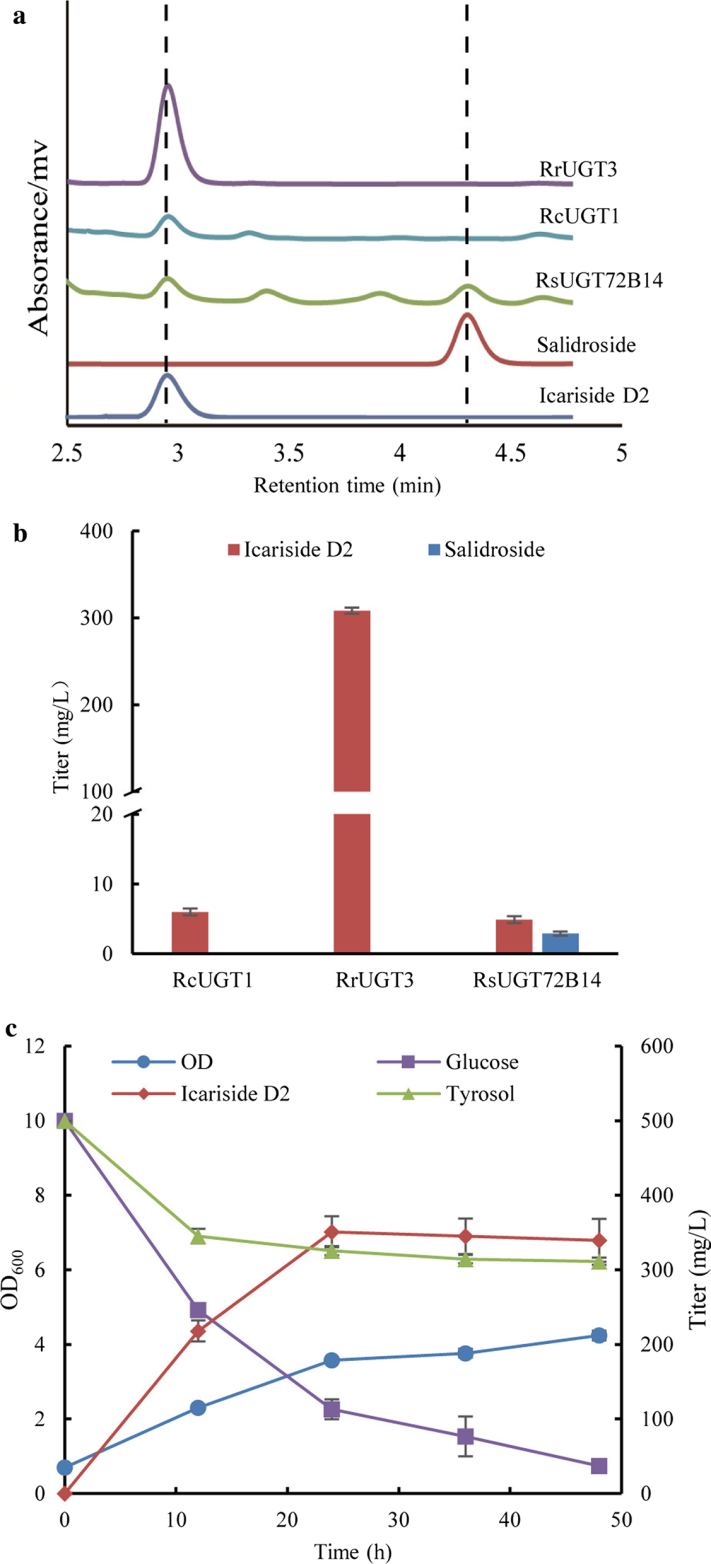
Heterologous biosynthesis of icariside D2 in *E. coli* BL21 (DE3) from tyrosol. **a** HPLC map of fermentation broth. **b** Production of icariside D2 by heterologous expression of *RcUGT1*, *RrUGT3,* and *RsUGT72B14*. **c** Time profile of icariside D2 fermentation from tyrosol by strain BMD2 expressing *RrUGT3*

To facilitate functional regio-selectivity of three UGT candidates, we performed the in vivo heterologous tyrosol glycosylation assay in *E. coli*. Codon-optimized genes *RcUGT1*, *RrUGT3,* and *RsUGT72B14* were synthesized and corresponding expression vectors were transferred to *E. coli* BL21 (DE3), generating strains BMD1, BMD2, and BMD3, respectively (Table 2). After fermentation supplemented with 500 mg/L of tyrosol, the supernatants of broth were subjected to HPLC analysis. The results showed that the expression of the *RrUGT3* and *RcUGT1* genes led to a new peak at retention time of 2.9 min, consistent with icariside D2 standard (Fig. 2a). The new compound had a molecular ion at *m*/*z* 318 ([M+NH4]^+^) corresponding to icariside D2 with molecular weight of 300 (Additional file 2: Fig. S2). In addition, the high-resolution HPLC–MS/MS showed that the main fragments of the new compound were identical with icariside D2 standard (Additional file 3: Fig. S3). It indicated that RrUGT3 and RcUGT1 exhibited glycosylation activities exclusively at phenolic position of tyrosol, selectively synthesizing icariside D2. However, in addition to icariside D2, RsUGT72B14 catalyzed the biosynthesis of salidroside at the same time, which agreed with the previous report [22].

As shown in Fig. 2b, three UGTs showed different capabilities of synthesizing icariside D2. Compared to RcUGT1 that generated 5.99 mg/L of icariside D2, RrUGT3 produced 307.3 mg/L of icariside D2, 51-fold higher than that of RcUGT1. Although RsUGT72B14 preferentially synthesized icariside D2 to salidroside, it produced minor titer of icariside D2. In view of the regio-selectivity and high productivity of RrUGT3 (Fig. 2c), it was employed for the production of icariside D2 in engineered *E. coli* in following study.

### Engineering *E. coli* monoculture for de novo production of icariside D2 from glucose

**Fig. 3:**
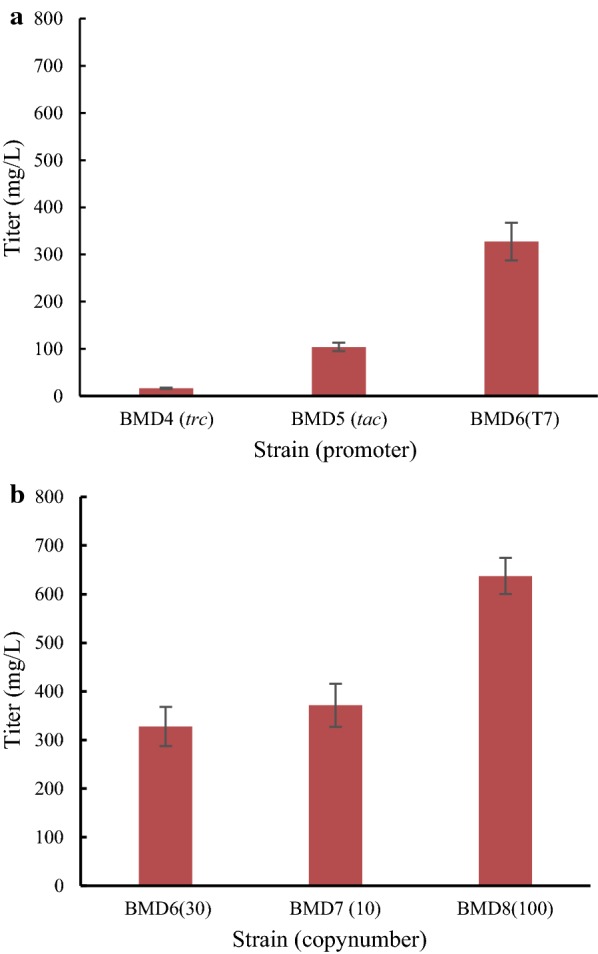
Fine tuning of the *RrUGT3* expression in *E. coli* BL21(DE3). **a** Altering the promoters. **b** Altering the copy numbers

Monoculture with single strain is a leading strategy for heterologous production of bio-based products. Here, we explored the production of icariside D2 using monoculture by balancing the gene expression involved in the biosynthetic pathway. The *trc* and *tac* promoters driving the heterologous expression cassettes are often utilized for their validity in various *E. coli* strains [23] and demonstrated to dramatically increase the titer of natural products [24]. To probe their potential on the biosynthesis of icariside D2, we cloned *RrUGT3* to pTrc99a, pGEX-6P-1, and pETDuet-1, and then introduced them into *E. coli* BL21 (DE3), generating strains BMD4, BMD5, and BMD6, respectively. As shown in Fig. 3a, strain BMD4 with the *trc* promoter led to the lowest icariside D2 titer of 16.43 mg/L and strain BMD5 with the *tac* promoter gave the moderate icariside D2 titer of 103.96 mg/L. Strain BMD6 with the T7 promoter led to the highest icariside D2 titer of 327.53 mg/L, twofold higher than that of the *tac* promoter, and 20-fold higher than that of the *trc* promoter, indicating that strong T7 promoter would benefit the biosynthesis of icariside D2 in *E. coli*.

Having confirmed the *RrUGT3* expression under the T7 promoter, we further investigated the effects of gene copy number which also played significant role in the heterologous expression and natural product titers [25, 26]. In addition to pETDuet-1 which is estimated about 30 copies in *E. coli*, we expressed the *RrUGT3* on pACYCDuet-1 (ca. 10 copies) and pRSFuet-1 (ca. 100 copies), respectively. As shown in Fig. 3b, strain BMD6 harboring pLXD6 (ETDuet-1 ori) and strain BMD7 harboring pLXD7 (ACYCDuet-1 ori) gave almost the same amount of icariside D2 (327.53 and 317.25 mg/L, respectively). Strain BMD8 harboring pLXD8 (RSFuet-1 ori) yielded much higher titer of 637.45 mg/L of icariside D2, about twofold of the former two counterparts. The significant advantage of BMD8 indicated that high copy number would facilitate the icariside D2 production. The same behaviors were reported in the production of natural products like hydroxytyrosol [27] and 4-hydroxymandelic acid [28].

**Fig. 4:**
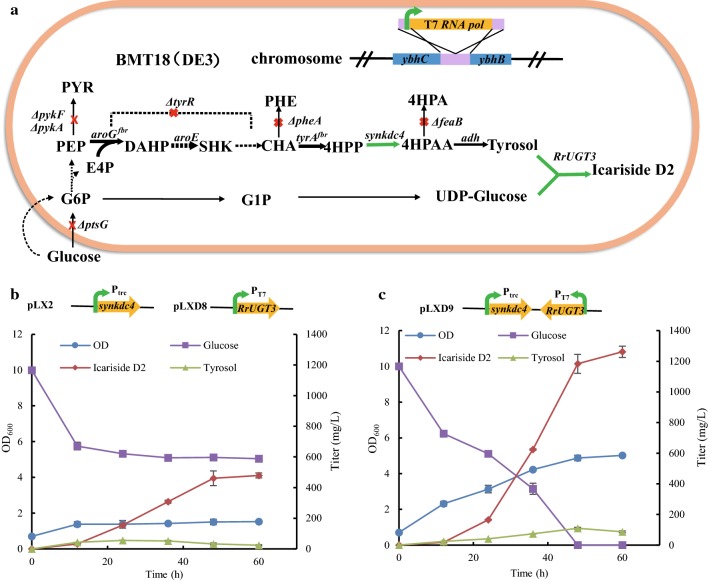
Metabolic engineering *E. coli* monoculture for production of icariside D2 from glucose. **a** The genetic characteristics of strain BMT18 (DE3). Red crosses indicated the deleted genes. Darken bold arrows indicated the overexpressed genes (*aroG*^*fbr*^, *aroE*, and *tyrA*^*fbr*^) in the chromosome. Green arrows indicated the heterologous genes. *G6P* glucose 6-phosphate, *G1P* glucose 1-phosphate, *E4P* erythrose 4-phosphate, *PEP* phosphoenolpyruvate, *PYR* pyruvate, *DAHP* 3-deoxyarabino-heptulonate 7-phosphate, *SHK* shikimate, *CHA* chorismic acid, *PHE* phenylalanine, *TYR* tyrosine, *fbr* feedback inhibition resistance. **b** Fermentation of strain BMD8, derived from BMT18 (DE3) harboring two plasmids pLX2 and pLXD8. **c** Fermentation of strain BMD10, derived from BMT18 (DE3) harboring one plasmid pLXD9

We previously engineered the tyrosol producing strain BMT21 by deleting the *feaB* gene of tyrosine overproducer BAK10 [29, 30] and heterologously expressing the synthetic *kdc4* (*synkdc4*) gene from *Pichia pastoris* GS115. The expression of *kdc4* gene was optimized from the inducible T7 promoter to the constitutive trc promoter in plasmid pLX2 (Table 2) [13]. For the functionality of T7 promoter, we integrating T7 RNA polymerase (T7 RNA pol) gene into the chromosome of strain BMT18 (*E. coli* BW25113 derivative) between the genes of *ybhb* and *ybhc* and constructed strain BMT18 (DE3) (Fig. 4a). For the de novo icariside D2 biosynthesis and attempt of combinatorial expression of *kdc4* and *RrUGT3*, pLX2 and pLXD8 were transferred to strain BMT18 (DE3), resulting in strain BMD9. Icariside D2 was successfully synthesized in strain BMD9 from glucose with the titer of 479.24 mg/L (Fig. 4b). However, cell growth was seriously retarded during the fermentation process, and the biomass did not increase after 12 h. The metabolic burden was a key factor leading to undesirable physiological changes, especially when the promoter strength and copy number increased for heterologous gene expression [31]. We speculated that two plasmids pLX2 and pLXD8 might cause *E. coli* cell to stop growing and tentatively combined the expression of *synkdc4* and *RrUGT3* in one plasmid pLXD9. As expected, not only was the cell growth recovered in the resulting strain BMD10 harboring plasmid pLXD9, but also a much greater production of icariside D2 was attained (Fig. 4c). The titer of icariside D2 in strain BMD10 rapidly increased from 24 h and ultimately reached 1.26 g/L at 48 h, 2.5-fold of strain BMD9. It suggested that balancing the promoter strength and copy number of gene expression could be an efficient approach for alleviating the plasmid-born metabolic burden to provide ample pathway efficiency [25].

### Engineering *E. coli*–*E. coli* coculture for production of icariside D2 from glucose–xylose mixture

**Fig. 5:**
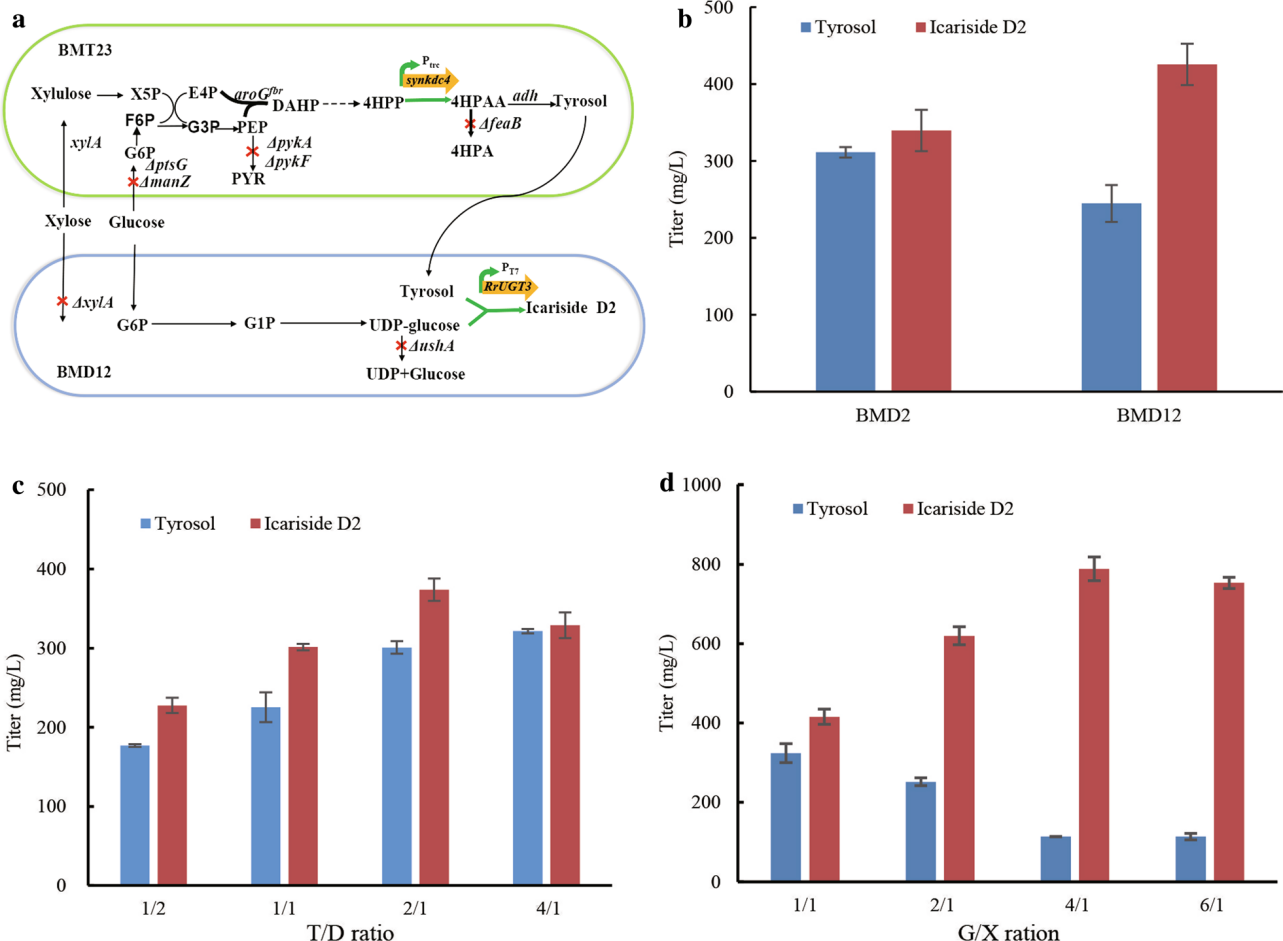
The *E. coli*–*E. coli* coculture for production of icariside D2 using glucose–xylose mixture. **a** The *E. coli*–*E. coli* coculture strategy for production of icariside D2. Strain BMT23 produced tyrosol preferentially using xylose, while strain BMD12 synthesized icariside D2 using glucose. *X5P* xylulose 5-phosphate, *F6P* fructose 6-phosphate, *G3P* glyceraldehyde 3-phosphate. **b** Effect of the *ushA* gene on icariside D2 biosynthesis. The *ushA* gene was deleted in strain BMD12 and not in strain BMD2. Fermentation was done with supplementation of 500 mg/L tyrosol. **c** Optimization of icariside D2 production by varying the inoculation ratio of BMD12 to BMT23 with the G/X ratio of 1/1. **d** Optimization of icariside D2 production by varying the ratio of glucose to xylose with the initial T/D ratio of 2/1

Plant-derived lignocellulose from the agricultural residues and energy crops is the most abundant renewable resource, with glucose and xylose as the major sugars in the pretreated hydrolysate [32]. Microbial coculture system as an emerging strategy of synthetic biology offers a promising platform for sustainable production of biofuels [33, 34], natural products [13, 35], and chemical bulks [36] using lignocellulosic sugar mixture as carbon sources. We previously constructed tyrosol overproducer BMT23 that utilized xylose preferentially with disrupted glucose uptake system, and used it for high production of salidroside in *E. coli*–*E. coli* coculture [13]. Here, to broaden fermentative carbon sources, we employed strain BMT23 as tyrosol donor, and engineered strain BMD12 as UDP-glucose supplier. We attempted to construct an *E. coli*–*E. coli* coculture for the sustainable production of icariside D2 from glucose–xylose mixture (Fig. 5a).

The supply of UDP glucose is often believed to be a limiting factor for high production of glycosides [15]. The UDP-sugar hydrolase encoded by the *ushA* gene would degrade the intracellular UDP glucose and impede the glycoside biosynthesis [37], and thus, we deleted the *ushA* gene of *E. coli* BL21 (DE3) to increase the UDP-glucose pool. We further deleted the *xylA* gene encoding xylose isomerase and constructed UDP-glucose overproducer BMD11 that utilized glucose exclusively. To test the availability of UDP glucose in strain BMD11, pLXD2 containing the *RrUGT3* gene was transferred to strain BMD11, giving rise to strain BMD12. As shown in Fig. 5b, when 500 mg/L of tyrosol was supplemented to the medium, strain BMD12 produce 425.56 mg/L of icariside D2, 25.5% higher than that without deletion of the *ushA* gene (339.55 mg/L), indicating the benefit of increasing the intracellular UDP-glucose supply for glycoside production. Thus, BMD12 was employed to engineer the coculture system with BMT23 to synthesize icariside D2 using glucose–xylose mixture.

For testing and balancing the icariside D2 biosynthetic pathway in the BMT23–BMD12 coculture, we first explored the inoculation ratios which were typical and significant strategy for optimizing the coculture [38]. To this end, different ratios of BMT23 to BMD12 (defined as T/D ratio) from 1/2 to 4/1 were inoculated when supplemented with 5 g/L of glucose and 5 g/L of xylose. As shown in Fig. 5c, the accumulation of tyrosol and icariside D2 was increased with the increase of T/D ratio, while the highest icariside D2 titer was achieved at T/D ratio of 2/1, and further enlargement of the inoculum of BMT23 hindered the conversion of tyrosol to icariside D2. Second, we investigated sugar mixture with different ratios of glucose to xylose (defined as G/X ratio) at the optimized T/D ratio of 2/1. As shown in Fig. 5d, when the G/X ratio was increased, tyrosol accumulation was declined and icariside D2 production increased quickly until the G/X ratio at 4/1, where the icariside D2 titer peaked at 788.21 mg/L. Further increasing the G/X ratio to 6/1 inhibited the icariside D2 production.

**Fig. 6:**
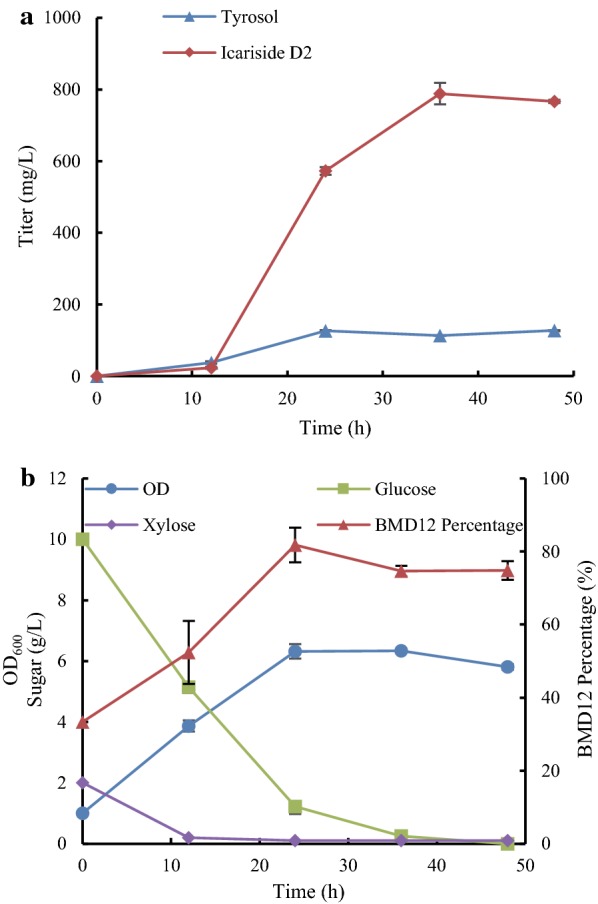
Fermentation dynamics of BMT23–BMD12 coculture with the G/X ratio of 4/1 and the initial T/D ratio of 2/1. **a** Time profile of icariside D2 and tyrosol production of batch fermentation. **b** Time profile of the overall cell density, sugar consumption, and BMD12 population percentage

The fermentation of sugar mixture functioned well in the *E. coli*–*E. coli* coculture system for the biosynthesis of icariside D2 in terms of smooth catabolism of glucose and xylose and considerable product titer. As shown in Fig. 6a, b, during the fermentation process of the BMT23–BMD12 coculture with the T/D ratio of 2/1 and the G/X ratio of 4/1, the glucose and xylose were simultaneously consumed and xylose was depleted rapidly at 12 h. The titer of icariside D2 increased tremendously from 12 h and achieved the maximum at 36 h with small amount of tyrosol (113.78 mg/L) remaining. Notably, we observed that the population composition in the coculture displayed a highly dynamic change after the inoculation. Strain BMD12 percentage in the coculture experienced a rapid increase from the initial 33.3% to 81.77% at 24 h and maintained about 74.82% throughout the fermentation process.

Maintaining microbial community stability and robustness is the key issue in engineered coculture system [39]. In this study, we stabilized the coculture by adjusting the inoculation ratio of two constituent strains and employing two diverse carbon sources for the cell growth of each strain. The rapid increase subpopulation of BMD12 in the first half of the fermentation was at least partially contributed by the faster growth of strain BMD12 than BMT23. The subpopulation of the BMD12 stabilized at approximately 74.82% after 24 h and efficiently converted tyrosol to icariside D2. It indicated that the higher inoculation subpopulation of BMT23 compensated its growth disadvantage over BMD12 and facilitated the stability of the BMT23–BMD12 coculture to balance the strength of icariside D2 biosynthetic pathway. Like BMD12 in our study here, it is common that one strain dominates the population in the artificial cocultures [35, 40], and it represents an efficient approach for balancing metabolic pathway strength in synthetic biology researches.

### Fed-batch fermentation of icariside D2

To investigate the long-term efficiency and stability of the icariside D2 production in engineered monoculture and coculture system, we performed the fed-batch fermentation in shake flask using sole glucose and glucose–xylose mixture, respectively.

**Fig. 7:**
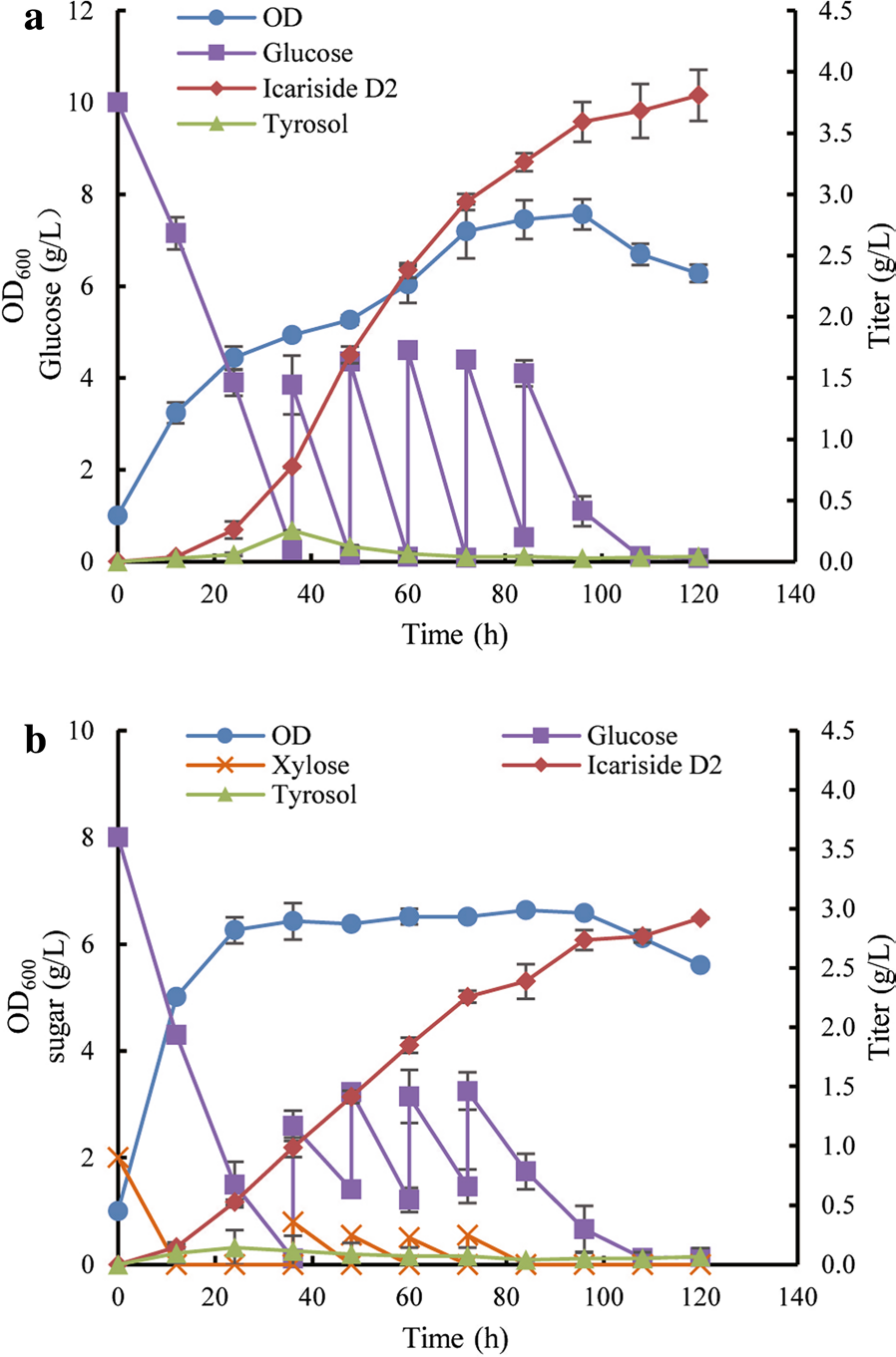
Fed-batch fermentation of **a** BMD10 monoculture using sole glucose and **b** BMT23–BMD12 coculture using glucose–xylose mixture in shake flask

For the fed-batch fermentation of strain BMD10 monoculture, 4 g/L of glucose was added every 12 h from 36 to 84 h. As shown in Fig. 7a, fed glucose was consumed quickly, and supported continuous cell growth before 72 h. Accordingly, icariside D2 was greatly accumulated from 24 to 120 h with the final titer of 3.80 g/L. Even in the stationary growth stage, strain BMD10 exhibited the efficient productivity. There was only a transient accumulation of precursor tyrosol at 36 h (255.43 mg/L), which was then converted promptly to icariside D2 after first glucose feeding and maintained at a very low level throughout the fermentation process.

Comparatively, coculture system of strains BMT23 and BMD12 exhibited different behaviors on cell growth and sugar consumption. As shown in Fig. 7b, xylose was depleted at 12 h, and glucose was used up at 36 h. After that, glucose–xylose mixture with G/X ratio of 4/1 was fed every 12 h from 36 to 72 h. The cells grew exponentially in first 24 h and then experienced a long stationary growth stage. During the fermentation process, with the utilization of sugar mixture, the production of icariside D2 linearly increased to the final titer of 2.92 g/L at 120 h. Tyrosol accumulation was also maintained at a very low level. The dynamics of BMD12 percentage was quite alike with that in batch fermentation, confirming that the coculture system was compatible for production of icariside D2.

To estimate the fermentation behaviors of the BMD10 monoculture and BMT23–BMD12 coculture, the biomass, growth rate, titer, yield, and productivity are summarized in Table 1. Production of icariside D2 in the BMD10 monoculture was much more coupled with cell growth than that in the BMT23–BMD12 coculture, which produced large amount of icariside D2 in the stationary stage. The BMD10 monoculture showed great capability of icariside D2 production with titer of 3.59 g/L and specific productivity of 37.39 (mg/(L·OD)) in 96 h, 31.50% and 31.52% higher than the BMT23–BMD12 coculture (Table 1), respectively. On the other hand, the BMT23–BMD12 coculture exhibited higher mole yield and mass yield, indicating more efficient conversion from sugar mixture to icariside D2 than the BMD10 monoculture from sole glucose. Taking together, these two strategies exhibited great potential for the efficient microbial production of icariside D2.

**Table 1:** The icariside D2 production using sole glucose and glucose–xylose mixture

Parameter	BMD10 monoculture	BMT23–BMD12 coculture
Carbon source consumed (g/L)	30.20 glucose	16.83 glucose and 4.40 xylose
Maximum OD	7.56	6.58
Growth rate (OD/h)	0.10	0.27
Titer (g/L)	3.59	2.73
Specific productivity (mg/(L·OD))	37.39	28.43
Yield (mol/mol of sugar)	0.071	0.074
Mass yield (%)	11.89	12.86

Values at 96 h were employed, except for the OD maximum and corresponding growth rates that were achieved at 72 h in monoculture and 24 h in coculture, respectively

## Conclusions

Natural glycosides have distinct pharmacological properties including antibiotic, anticancer, and antioxidant activities with high solubility and stability [41]. Microbial production of high-value natural glycosides as an alternative to replace direct isolation from plants has already attracted extensive attention in pharmaceutical and nutraceutical studies. The great advance of synthetic biology facilitates the engineering of microbial cell factories to biosynthesize various natural compounds. The UGTs attach sugar residues to aglycones and often represent the metabolic bottleneck in microbial production of glycosides. Fine-tuning expression patterns of UGTs could balance the heterologous biosynthetic pathway of glycosides. Here, we showed that combinatorial expression of *synkdc4* and *RrUGT3* in one middle copy-number plasmid was better than separate expression in middle and high ones for production of icariside D2 from glucose. Thus, our results exhibited that engineered strain BMD10 monoculture was an excellent case for great capability of glycoside production from sole glucose.

Microbial cocultures have proven to be attractive routes to microbial production [42] for the instinctive advantages in dividing metabolic burden [14], compartmentalizing special bioreactions [43], decreasing byproducts formation [44], and especially coutilizing lignocellulose [31]. We distributed the biosynthetic pathway of icariside D2 to two *E. coli* strains, and engineered an *E. coli*–*E. coli* coculture where the tyrosol producer BMT23 preferentially utilized xylose and icariside D2 producer BMD12 consumed glucose exclusively. The BMT23–BMD12 coculture was competent for sustainable production of icariside D2 from glucose–xylose mixture and comparable to the BMD10 monoculture. *E. coli*–*E. coli* coculture by balancing the biosynthetic pathways provides the promising potential for efficient production of natural and non-natural products.

## Methods

### Strains, plasmids, and reagents

The bacterial strains and plasmids used in this study are listed in Tables 2, 3. Icariside D2 (98% purity) and tyrosol (98% purity) were purchased from Dingguo Biotech (Tianjin, China). Methanol and acetate (HPLC grade) were purchased from Concord Tech (Tianjin, China). The ClonExpress II One-Step Cloning Kit was obtained from Biomed (Beijing, China) and DNA Polymerase of Phanta Super Fidelity and Rapid *Taq* Master Mix from Vazyme (Nanjing, China). PCR primers are synthesized by GENEWIZ (Suzhou, China) and listed in Additional file 4: Table S1.

**Table 2:** Bacterial strains and plasmids used in this study

Strain	Characteristics	Source
*E. coli* BW25113	*lacI*^q^*rrnB*_T14_Δ*lacZ*_WJ16_ *hsdR514* Δ*araBAD*_AH33_ Δ*rhaBAD*_LD78_	NBRP-*E. coli* at NIG
BAK5	BW25113∆*ptsG*::∆*tyrR*::∆*pykA*:: *pykF*::∆*pheA*::FRT	[29]
BAK10	BAK5*Δmao*-*paa cluster::*P_*lacUV5*_-*aroG*^*fbr*^-*tyrA*^*fbr*^-*aroE*	[30]
BMT18	BAK10 Δ*feaB*::FRT	[13]
BMT18(DE3)	BMT18 P_*lacUV5*_-T7 RNA pol	This study
BMT23	BAK10 Δ*feaB*::FRT Δ*manZ*::chl pLX2	[13]
BMD1	BL21(DE3) pLXD1	This study
BMD2	BL21(DE3) pLXD2	This study
BMD3	BL21(DE3) pLXD3	This study
BMD4	BL21(DE3) pLXD4	This study
BMD5	BL21(DE3) pLXD5	This study
BMD6	BL21(DE3) pLXD6	This study
BMD7	BL21(DE3) pLXD7	This study
BMD8	BL21(DE3) pLXD8	This study
BMD9	BL21(DE3) pLX2 pLXD8	This study
BMD10	BMT18(DE3) pLXD9	This study
BMD11	BL21(DE3) Δ*ushA*::kan Δ*xylA*::FRT	This study
BMD12	BMD11 pLXD2	This study

**Table 3:** Plasmids used in this study

Plasmid	Characteristics	Source
pKD46	Red recombinase expression vector; Amp^R^	[45]
pKD3	FRT (FLP recognition target) sites; Cm^R^	[45]
pKD4	FRT (FLP recognition target) sites; Kan^R^	[45]
pCP20	FLP expression vector; Amp^R^	[45]
pCDFDuet-1	pCDF ori with P_T7_; Sm^R^	Novagen
PGEX-6P-1	colE1 ori with P_tac_; Amp^R^	Novagen
pTrc99A	colE1 ori with P_trc_; Amp^R^	Novagen
pETDuet-1	colE1 ori with P_T7_; Amp^R^	Novagen
pACYCDuet-1	p15A ori with P_T7_; Cm^R^	Novagen
pRSFDuet-1	RSF ori with P_T7_; Kan^R^	Novagen
pLXD1	pCDFDuet-1 with RcUGT1	This study
pLXD2	pCDFDuet-1 with RrUGT3	This study
pLXD3	pCDFDuet-1 with RsUGT72B14	This study
pLXD 4	pTrc99A with RrUGT3	This study
pLXD 5	PGEX-6P-1 with RrUGT3	This study
pLXD 6	pETDuet-1 with RrUGT3	This study
pLXD 7	pACYCDuet-1 with RrUGT3	This study
pLXD 8	pRSFDuet-1 with RrUGT3	This study
pLX2	pTrc99a (with deletion of lacI sequence and change from ampR to strR) with synkdc4	[13]
pLXD9	pLX2 with T7-RrUGT3	This study

### Plasmids and strains’ construction

Gene encoding glycosyltransferase RsUGT73B6 (GenBank: AY547304), RrUGT17 (GenBank: MF674542.1), YjiC (GenBank: AAU40842), RcUGT1 (GenBank: MH299424.1), RrUGT3 (GenBank: MF674528.1), and RsUGT72B14 (GenBank: EU567325) were synthesized by GenScript (Nanjing, China) with codon optimization for *E. coli*. Their nucleic acid sequences are listed in Additional file 5: Table S2.

ClonExpress II One-Step Cloning Kit was utilized to construct expression vectors. The codon-optimized *RcUGT1*, *RrUGT3*, and *RsUGT72B14* were cloned to pCDFDuet-1 to generate plasmids pLXD1, pLXD2, and pLXD3, respectively. Similarly, the *RrUGT3* gene was cloned into pTrc99a, pGEX-6P-1, pETDuet-1, pACYCuet-1, and pRSFuet-1 to generate plasmids pLXD4 to pLXD8, respectively. T7-*RrUGT3* sequence was amplified from pLXD2 using primers LXD9-5F1 and LXD9-3R1, and then ligated to the linearized pLX2 that was obtained using primers LXD9-5F2 and LXD9-3R2, resulting in plasmid pLXD9.

All in-frame gene deletion and gene integration strains were constructed using the classical λ Red homologous recombination method [45] and further confirmed by PCR. When deleting *ushA* gene in strain BMD12, kanamycin resistance fragment was amplified from pKD4 and used for the replacement of *ushA* gene. When deleting *manZ* gene in strain BMT23, chloramphenicol resistance fragment was amplified from pKD3 and used for the replacement of *manZ* gene. T7 RNA pol gene was amplified from chromosome of *E. coli* BL21 (DE3) using primers T7 RNA pol-5F and T7 RNA pol-3R and inserted into the chromosome of BMT18 between the *ybhB* and *ybhC* genes.

### Media and cultivation conditions

For strain maintenance and seed preparation, Luria–Bertani (LB) medium (10 g/L tryptone, 5 g/L yeast extract, and 10 g/L NaCl) was used. All batch fermentations were carried out in M9Y medium (17.1 g/L Na_2_HPO_4_·12H_2_O, 3.0 g/L KH_2_PO_4_, 0.5 g/L NaCl, 1.0 g/L NH_4_Cl, 5 mM MgSO_4_, 0.1 mM CaCl_2_, and 1 g/L yeast extract). Carbon source of sole glucose, or mixture of glucose and xylose with desired amounts was supplemented. Appropriate amounts of antibiotics (20 μg/mL chloramphenicol, 100 μg/mL ampicillin, 30 mg/L kanamycin, and 30 μg/mL streptomycin) were added when necessary. 0.1 mM IPTG was supplemented into the medium when needed.

Fermentation experiments were started with an overnight LB culture followed by a dilution of 1:100 to into 50 mL of fresh LB medium, cultivated for 4–6 h at 220 rpm and 37 °C. Cells were then harvested and transferred to 250 mL of shake flask with 50 mL of M9Y medium. For the optimization of *RrUGT3* expression, 500 mg/L of tyrosol was added. Cultures were incubated at 30 °C and 200 rpm for batch fermentation and fed-batch fermentation. The fermentation experiments were conducted in triplicates and data were shown as the mean ± S.D.

### Coculture system analysis

To measure the population of BMT23 in coculture system, BMT23 was engineered to possess the resistance to chloramphenicol. The fermentation broth were periodically withdrawn and centrifuged, and the cell pellets with appropriate dilutions were plated on LB plates containing chloramphenicol. The plates were then incubated at 37 °C for 12 h and colony-forming units (CFU) on each plate (50–500 colonies each plate) were manually counted.

Strain BMD12 grew normally in the liquid M9Y with or without IPTG induction. However, after IPTG induction, most of BMD12 cells did not form colonies on the solid LB plates. The CFU of BMD12 in the coculture could not be measured directly by colony counting method. Possibly, the expression of RrUGT3 in the liquid BMD12 by IPTG induction might be toxic for cell growth on the solid LB plates. BMT23 grew normally either in liquid medium or on solid medium with or without the IPTG induction. The percentage of BMD12 in the BMT23–BMD12 coculture could be estimated indirectly using the total OD of the coculture and OD of BMT23 subpopulation that was calculated through the calibration curve from the CFU (Additional file 6: Fig. S4).

### Biomass and metabolite analysis

Cell optical density (OD) was measured at 600 nm with TU-1810 spectrophotometer. Residual glucose and xylose were quantified by Waters 1515 HPLC system, equipped with a Bio-Rad HPX-87H column and a refractive index detector (Waters 2414, Milford, USA). Isocratic elution through the column was conducted using 5 mM of sulfuric acid at 0.6 mL/min and 65 °C.

Quantification of tyrosol and icariside D2 was carried out using Agilent 1200 HPLC system equipped with a C18 column (150 * 4.6 mm with a particle size of 5 μm, Bonna-Agela, China) and a PDA detector (Agilent). After fermentation, the broth samples were centrifuged, and 10 μL of cell-free supernatants was filtered through 0.22 μm pore-sized syringe filter before being measured under room temperature with a mobile phase (20% methanol, 80% water, and 0.1% acetate) at 1 mL/min. The tyrosol and icariside D2 were measured at 225 nm. The structure of icariside D2 was further analyzed using high-resolution LC–MS/MS [Synapt G2-Si Q-TOF mass spectrometer coupled with an ACQUITY UPLC system (Waters, USA)] under positive-ion mode. All of the HPLC analysis were quantified using a five-point calibration curve and the *R*^2^ coefficient for the calibration curve was higher than 0.99.

## Supplementary information

**Additional file 1: Fig. S1.** Production of tyrosol glycosides by heterologous expression of regio-promiscuous UGTs of RsUGT73B6, RrUGT17 and Yjic in *E. coli* BL21 (DE3).

**Additional file 2: Fig. S2.** The LC–MS of the (A) icariside D2 standard and (B) fermentation supernatant sample of strain BMD2. Strain BMD2 produced a new product that has identical retention time with standard icariside D2 and the primary ion fragment at *m*/*z* 318 ([M+NH4]^+^) corresponds to icariside D2 with molecular weight of 300.

**Additional file 3: Fig. S3.** The high resolution LC–MS/MS of the (A) icariside D2 standard and (B) fermentation supernatant sample of strain BMD2. The main fragments of new compound were identical with icariside D2 standard.

**Additional file 4: Table S1.** The main primers used in this study.

**Additional file 5: Table S2.** Nucleotide sequences of codon optimized genes.

**Additional file 6: Fig. S4.** Calibration curve of BMT23 obtained by counting colony forming units at different OD_600_ without the IPTG.

## Data Availability

The data sets used and/or analyzed during the current study are available from the corresponding author on reasonable request.

## Chemicals

CHA: chorismic acid
DAHP: 3-deoxyarabino-heptulonate 7-phosphate
E4P: erythrose 4-phosphate
F6P: fructose 6-phosphate
G3P: glyceraldehyde 3-phosphate
4HPA: 4-hydroxyphenylacetate
4HPAA: 4-hydroxyphenylacetaldehyde
4HPP: 4-hydroxyphenylpyruvate
G1P: glucose 1-phosphate
G6P: glucose 6-phosphate
PEP: phosphoenolpyruvate
PHE: phenylalanine
PYR: pyruvate
SHK: shikimate
TYR: tyrosine
UDP glucose: uridine 5, 9-diphosphoglucose
X5P: xylulose 5-phosphate

## Enzymes

ADH: alcohol dehydrogenase
AT: aminotransferase
KDC: keto acid decarboxylase
TDC: tyrosine decarboxylase
TYO: tyramine oxidase
UGT: uridine diphosphate glycosyltransferase
