# Supplementary figures and images for: Metabolic engineering Escherichia coli for efficient production of icariside D2
Source: Biotechnol Biofuels. 2019 Nov 6;12:261. doi: 10.1186/s13068-019-1601-x (PMC6833136; doi:10.1186/s13068-019-1601-x)

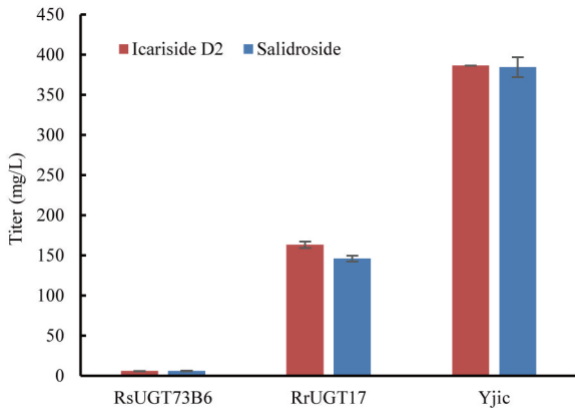

Supplement: Supplementary file 1 — Additional file 1: Fig. S1. Production of tyrosol glycosides by heterologous expression of regio-promiscuous UGTs of RsUGT73B6, RrUGT17 and Yjic in E. coli BL21 (DE3). [file 13068_2019_1601_MOESM1_ESM.pdf]

A

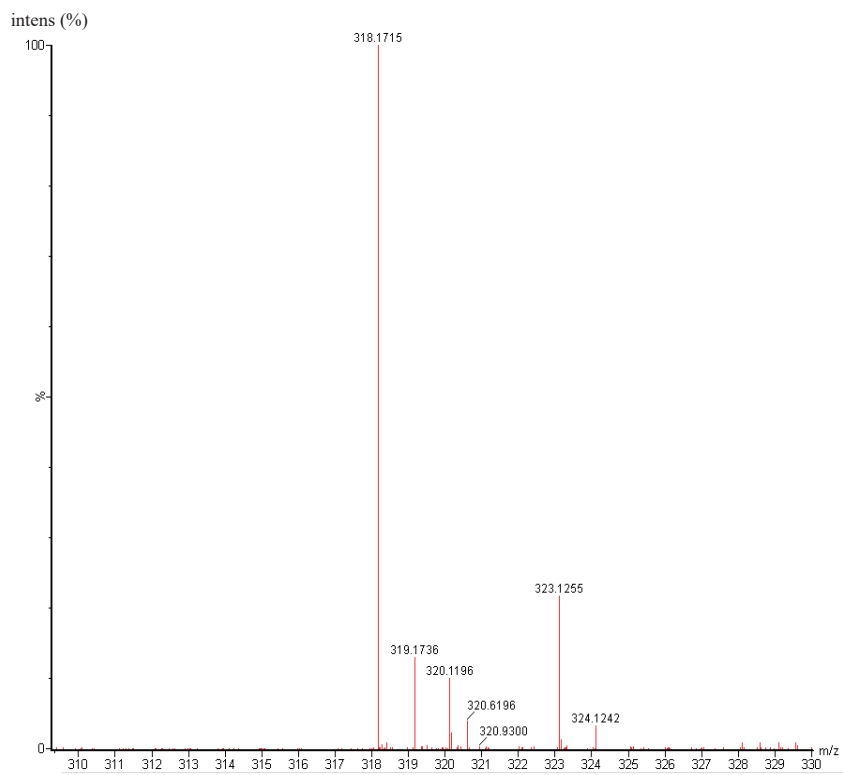

B

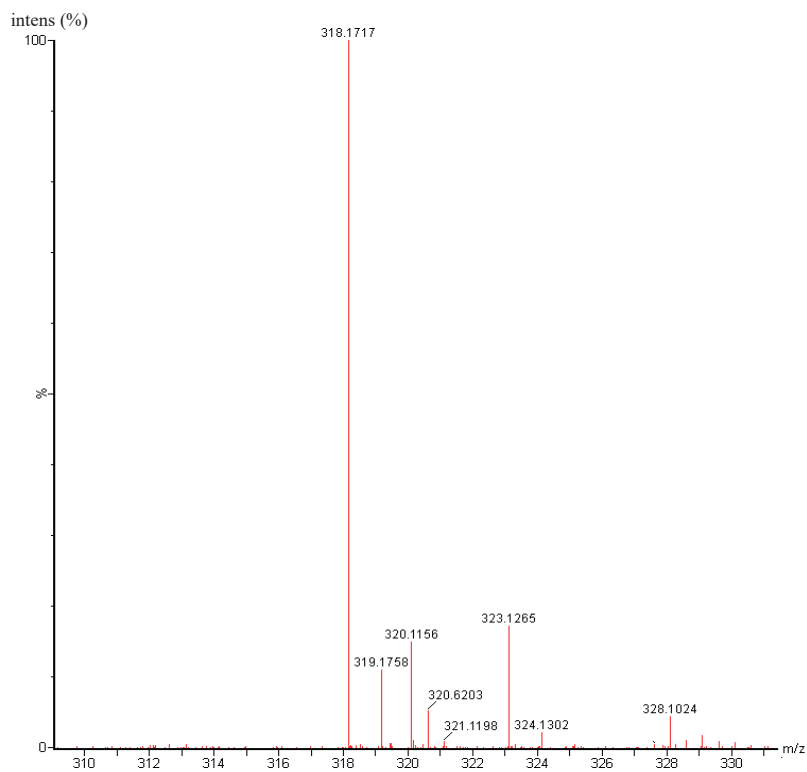

Supplement: Supplementary file 2 — Additional file 2: Fig. S2. The LC–MS of the (A) icariside D2 standard and (B) fermentation supernatant sample of strain BMD2. Strain BMD2 produced a new product that has identical retention time with standard icariside D2 and the primary ion fragment at m/z 318 ([M+NH4]+) corresponds to icariside D2 with molecular weight of 300. [file 13068_2019_1601_MOESM2_ESM.pdf]

A

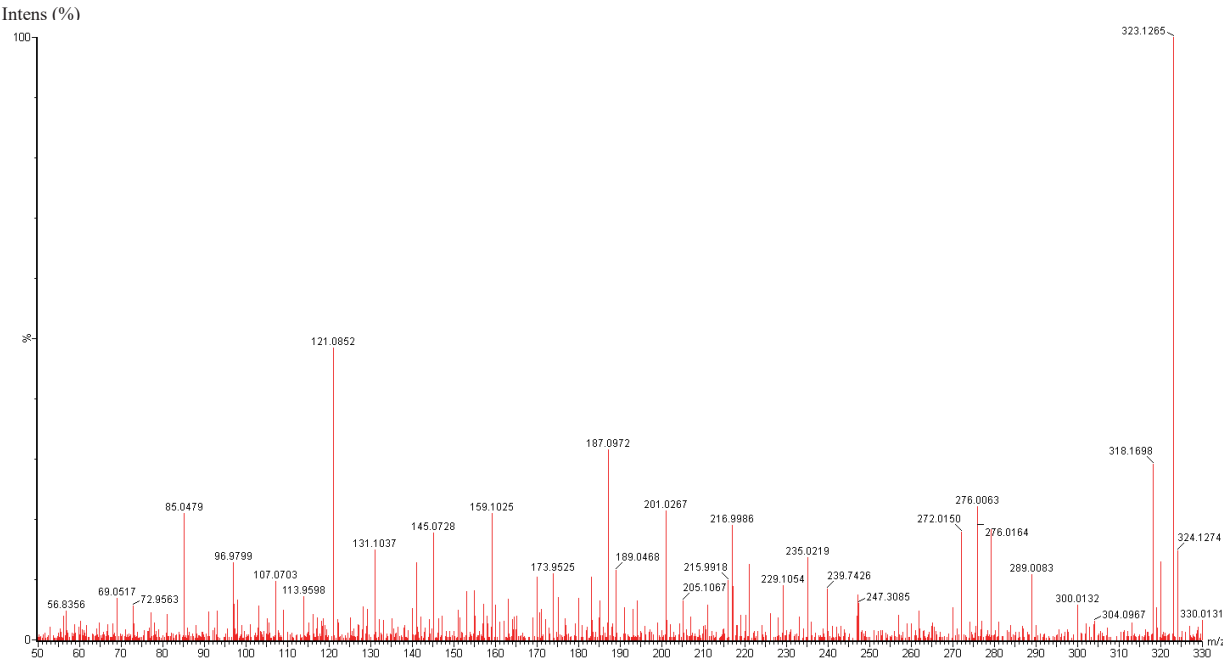

B

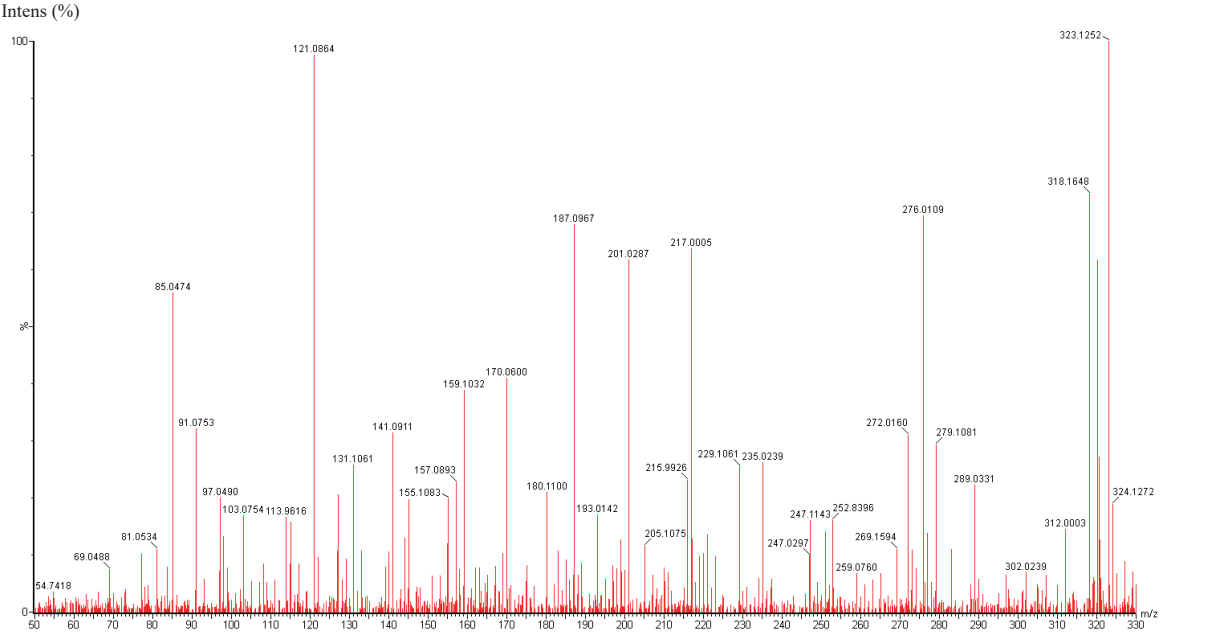

Supplement: Supplementary file 3 — Additional file 3: Fig. S3. The high resolution LC–MS/MS of the (A) icariside D2 standard and (B) fermentation supernatant sample of strain BMD2. The main fragments of new compound were identical with icariside D2 standard. [file 13068_2019_1601_MOESM3_ESM.pdf]

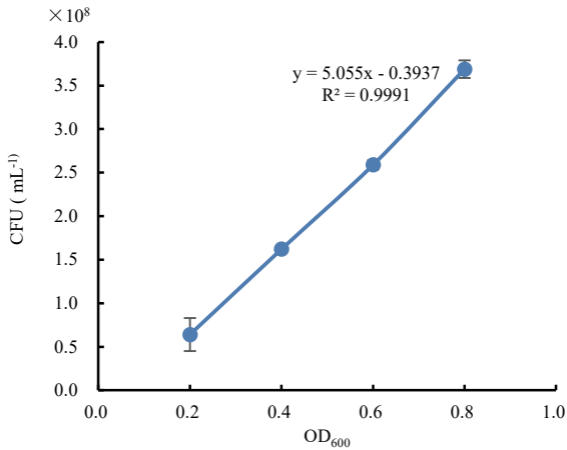

Supplement: Supplementary file 6 — Additional file 6: Fig. S4. Calibration curve of BMT23 obtained by counting colony forming units at different OD600 without the IPTG. [file 13068_2019_1601_MOESM6_ESM.pdf]
