# Supplementary material for: Metabolic engineering Escherichia coli for efficient production of icariside D2
Source: Biotechnol Biofuels. 2019 Nov 6;12:261. doi: 10.1186/s13068-019-1601-x (PMC6833136; doi:10.1186/s13068-019-1601-x)
Supplement: Supplementary file 4 — Additional file 4: Table S1. The main primers used in this study. [file 13068_2019_1601_MOESM4_ESM.docx]

**Table S1. The main primers used in this study.**

| Primer | Sequence |
| --- | --- |
| **Gene overexpression** |  |
| 5F-1 | CTCGAGTCTGGTAAAGAAACC |
| 3R-1 | GAGATCTGCCATATGTATATCTC |
| UGT72b14-5F | GATATACATATGGCAGATCTC ATGGCTGGTTCTGGTACTGG |
| UGT72b14-3R | GGTTTCTTTACCAGACTCGAGTTAGTGTTTAACAGAAGAACGC |
| RrUGT3-5F | GATATACATATGGCAGATCTCATGTCTGGCACCCCACACAT |
| RrUGT3-3R | GGTTTCTTTACCAGACTCGAGTTAATGCTTCATAGAAGAA |
| RcUGT1-5F | GATATACATATGGCAGATCTCATGGACAGCGATAGCCGTCC |
| RcUGT1-3R | GGTTTCTTTACCAGACTCGAGTTAGCTCAGCGGAACACGAC |
| pTrc-5F | GGATCCTCTAGAGTCGACCTG |
| pTrc-3R | TTCCATGGTCTGTTTCCTGTGTGAAATTCCACACATTATACGAGCCGG |
| Trc-UGT3-5F | CAGGAAACAGACCATGGAAATGTCTGGCACCCCACACATCG |
| Trc-UGT3-3R | CAGGTCGACTCTAGAGGATCCTTAATGCTTCATAGAAGAAC |
| pGEX-5F | TCACAGCTTGTCTGTAAGCG |
| pGEX-3R | GAATACTGTTTCCTGTGTGAAATTG |
| pGEX-UGT3-5F | TCACACAGGAAACAGTATTCATGTCTGGCACCCCACACAT |
| pGEX-UGT3-3R | CGCTTACAGACAAGCTGTGATTAATGCTTCATAGAAGAAC |
| pLXD9-5F1 | ATCCTGACGGATGGCCTTTTCAAAAAACCCCTCAAGACCC |
| pLXD9-3R1 | AAAGAGTTTGTAGAAACGCATCGAACAGAAAGTAATCGTA |
| pLXD9-5F2 | TGCGTTTCTACAAACTCTTTT |
| pLXD9-3R2 | AAAAGGCCATCCGTCAGGAT |
| ybhC-5F | ATCAA GGGAA AGCCC AATCT |
| ybhC-3R | AAGTT AGTAT AAAAA AGCAG |
| T7 RNA pol-5F | TCACT CATTA GGCAC CCC |
| T7 RNA pol-3R | GTATC AAGGT ATTTT ATGCG |
| T7-chl-5F | CTGCTTTTTTATACTAACTTGTAGGCTGGAGCTGCTTC |
| T7-chl-3R | TTTACCTTCCCGTTTCGCTCGTATCAAGGTATTTTATGCG |
| ybhB-5F | GAGCG AAACG GGAAG GTAAA |
| ybhB-3R | AGAAA GGAGG GTTCA TGAAA |
|  |  |
| **Gene deletion** |  |
| ushA-up-5F | ATAGCTGCTGATGCTCGCCAT |
| ushA-up-3R | ATCAGGTCAGGGAGAGAAGTTGTAGGCTGGAGCTGCTTCG |
| ushA-kan-5F | CGAAGCAGCTCCAGCCTACAACTTCTCTCCCTGACCTGAT |
| ushA-kan-3R | AAACATCCGGCACTTTCGGAATTCCGGGGATCCGTCGACC |
| ushA-down-5F | GGTCGACGGATCCCCGGAATTCCGAAAGTGCCGGATGTTT |
| ushA-down-3R | TGGCATACCAACGCACGGTT |
| xylA-chl-5F | ATTACGACATCATCCATCACCCGCGGCATTACCTGATTATGGAGTTCAATGTGTAGGCTG GAGCTGCTTC |
| xylA-chl-3R | TGCCCGGTATCGCTACCGATAACCGGGCCAACGGACTGCACAGTTAGCCGATGGGAATTAGCCATGGTCC |
