# Supplementary material for: Metabolic engineering Escherichia coli for efficient production of icariside D2
Source: Biotechnol Biofuels. 2019 Nov 6;12:261. doi: 10.1186/s13068-019-1601-x (PMC6833136; doi:10.1186/s13068-019-1601-x)
Supplement: Supplementary file 5 — Additional file 5: Table S2. Nucleotide sequences of codon optimized genes. [file 13068_2019_1601_MOESM5_ESM.docx]

**Table S2. Nucleotide sequences of codon optimized genes.**

| *RsUGT73B6* | ATGGGTTCTGAAACCCGTCCGCTGTCTATCTTCTTCTTCCCGTTCATGGCTCACGGTCACATGATCCCGATGGTTGACATGGCTCGTCTGTTCGCTTCTCAGGGTGTTCGTTGCACCATCGTTACCACCCCGGGTAACCAGCCGCTGATCGCTCGTTCTATCGGTAAAGTTCAGCTGCTGGGTTTCGAAATCGGTGTTACCACCATCCCGTTCCGTGGTACTGAATTTGGTCTGCCGGACGGTTGCGAAAACCTGGACTCTGTTCCGTCTCCGCAGCACGTTTTCCACTTCTTCGAAGCTGCTGGTTCTCTGCGTGAACCGTTCGAACAGCTGCTGGAAGAACACAAACCGGACTGCGTTGTTGGTGACATGTTCTTCCCGTGGTCTACCGACTCTGCTGCTAAATTCGGTATCCCGCGTCTGGTTTTCCACGGTACTTCTTACTTCGCTCTGTGCGCTGGTGAAGCTGTTCGTATCCACAAACCGTACCTGTCTGTTTCTTCTGACGACGAACCGTTCGTTATCCCGGGTCTGCCGGACGAAATCAAACTGACCAAATCTCAGCTGCCGATGCACCTGCTGGAAGGTAAAAAAGACTCTGTTCTGGCTCAGCTGCTGGACGAAGTTAAAGAAACCGAAGTTTCTTCTTACGGTGTTATCGTTAACTCTATCTACGAACTGGAACCGGCTTACGCTGACTACTTCCGTAACGTTCTGAAACGTCGTGCTTGGGAAATCGGTCCGCTGTCTCTGTGCAACCGTGACGTTGAAGAAAAAGCTATGCGTGGTATGCAGGCTGCTATCGACCAGCACGAATGCCTGAAATGGCTGGACTCTAAAGAACCGGACTCTGTTGTTTACGTTTGCTTCGGTTCTACCTGCAAATTCCCGGACGACCAGCTGGCTGAAATCGCTTCTGGTCTGGAAGCTAGTGGTCAGCAGTTCATCTGGGTTATCCGTCGTATGTCTGACGACTCTAAAGAAGACTACCTGCCGAAAGGTTTCGAAGAACGTGTTAAAGACCGTGCTCTGCTGATCCGTGGTTGGGCTCCGCAGGTTCTGATCCTGGACCACCAGTCTGTTGGTGGTTTCGTTTCTCACTGCGGTTGGAACTCTACCCTGGAAGGTATCTCTGCTGGTCTGCCGATGGTTACCTGGCCGGTTTTCGCTGAACAGTTCTACAACGAAAAACTGCTGACCGAAGTTCTGAAAATCGGTGTTGCTGTTGGTGCTCGTAAATGGCGTCAGCTGGTTGGTGACTTCGTTCACAAAGACGCTATCCAGCGTGCTGTTCGTGAAATCATGGAAGGTGAAGAAGCTGAAGAACGTCGTATCATCGCTCGTCAGATGGGTAAAATGGCTAAACGTGCTGTTGAAAAAGACGGTTCTTCTTGGACCAACCTGAACAACCTGCTGCAAGAACTGAAACTGAAAAAAGTTTAA |
| --- | --- |
| *RrUGT17* | ATGGGCTCTCTGCCGTCCACCAAATCCCATGCAGTTCTGGTTCCATACCCGGCCCAAGGCCACATCAACCCGTTCATGCAACTGGCCAAGCTGCTGCACTCTAAAGGTTTCCACATCACCTTCGTTAACAACGACCACAACCATCGCCGTCTGCTGCGTACCAAAGGTCATGATTTTGTTCAAGGTCTGGAAGGTCTGCGTTTTGAAGCTGTGCCGGATGGCCTGCCGCCATCTGACCGTGATGCCACTCAGGATGTTCCGAAGCTGACTGAATCTATTTACAACAAGAGCATGAACCAACCGTTCTCTGATCTGCTGCAACGTCTGAACTCTACCCCGGGTTCCCCGCCGGTTACTTGTGTTATCTCCGATGTTGCCATGTTTTTTGCTTGGGACGTGGCGGATGAGCTGGGCATCCCGAACGTTCAGTTTTGGACCGCTTCTGCTTGTGGCCTGCTGGGTTACCTGCAATATGATGAGCTGCTGCGTCGTGCCATCGTTCCATTCAAAGATGAAAACTTCATGACCGATGGTTCTCTGGAGGCTCTGATTGACTGGATTCCGGGCATGCCGAACATGCGTCTGAAGGACCTGCCATCGTTCATGCGTACCACCAGCCCGGACGACGTGCTGTTCAACTACCTGCGTACCATCACCACCAAAGCTCTGAAATCCTCTGCCCTGCTGCTGAACACCTTTGATGATTTTGAACATGAAGTAGTTGAAGAGATGAAGAAAATGCAACCAAACATCTTCCTGGGTGGTCCACTGAACATGCTGCTGCGTCACACCTCTAAAACTGAAATCACCTCCCTGACCACCTCTCTGTGGAAAGAGGACACTCATTGTCTGGAATGGCTGGACAAGCAAGAACCGGAGTCTGTGGTATACATCAACTACGGTTCTGTGACCATCATGTCTGATCACCATCTGAACGAGTTTGCTTGGGGTCTGGCTAACAGCAAGCATCCGTTCCTGTGGATCGTCCGTCCGGATGTTGTGCGTGGCGAGTCTGGTACTCTGCCGAAGGAGTTTTATGATGAGATCAAGGACCGTGGTCTGATCACCAGCTGGTGTCCGCAACCAGAGGTGCTGAAACATCCATCCGTAGGTGTATACCTGACCCATTGTGGTTGGAACTCTATCACCGAGTCTGTGGCCGGTGGTGTGCCACTGATGTGCTGGCCGTTTTTCGCTGAGCAACAGACCAACAGCCGTTTCGCGTGTACCGTGTGGGGCACTGGTGTGGAGGTGAACGCGGATGTGAAGCGTGAGGAGCTGGCGGAACAAGTGATGGAGATGCTGGAAGGTAAGCGTGGTCAAGAGCTGCGTAAAAACGCTAAGGAGTGGCGTCGTAAGGCGGAGGAGGCGACCGACATTGGCGGTTCTGCGTACGCTGACTTCGATCGTTTTATGGAAAAAGTGGTTCAGTTTAGCGTTTAA |
| *YjiC* | ATGGGTCATAAACATATCGCGATTTTTAACATTCCGGCTCACGGCCATATTAACCCGACCCTGGCTCTGACCGCAAGCCTGGTTAAACGCGGTTATCGTGTAACCTATCCGGTGACCGATGAGTTTGTGAAGGCTGTTGAGGAAACCGGTGCAGAGCCGCTGAACTACCGCTCTACCCTGAACATCGATCCGCAGCAAATTCGTGAGCTGATGAAAAACAAAAAAGATATGTCTCAGGCTCCGCTGATGTTTATCAAAGAAATGGAGGAGGTTCTGCCGCAGCTGGAAGCGCTGTATGAGAACGACAAGCCGGACCTGATACTCTTTGACTTCATGGCAATGGCGGGTAAACTGCTGGCTGAGAAGTTTGGTATCGAGGCGGTTCGCCTGTGTTCTACCTATGCACAGAACGAACATTTTACCTTCCGTTCCATTTCTGAAGAGTTTAAGATCGAGCTGACCCCGGAGCAAGAGGATGCTCTGAAAAACTCTAACCTGCCGTCTTTTAACTTTGAGGATATGTTCGAGCCGGCAAAACTGAACATTGTTTTTATGCCGCGTGCTTTTCAGCCGTACGGCGAAACCTTTGATGAGCGTTTCTCTTTTGTTGGTCCGTCTCTGGCCAAACGCAAGTTTCAGGAAAAAGAAACCCCGATTATTTCTGACAGCGGCCGTCCGGTTATGCTGATCTCTCTGGGTACTGCGTTCAACGCCTGGCCGGAATTTTATCACATGTGCATCGAAGCATTCCGTGACACCAAGTGGCAGGTTATCATGGCTGTTGGCACCACCATCGATCCGGAATCGTTTGATGACATCCCGGAGAACTTTTCTATTCATCAGCGCGTTCCGCAGCTGGAGATCCTGAAGAAAGCGGAGCTGTTCATCACCCACGGTGGTATGAACTCTACTATGGAAGGTCTGAACGCCGGTGTACCGCTGGTTGCCGTTCCGCAAATGCCGGAACAGGAAATCACCGCCCGCCGCGTTGAAGAGCTGGGTCTGGGCAAGCATCTGCAACCGGAAGACACCACCGCAGCTAGTCTGCGTGAAGCCGTAAGCCAGACCGATGGTGACCCGCATGTTCTGAAACGTATCCAGGACATGCAAAAGCACATTAAACAAGCCGGTGGTGCCGAGAAAGCCGCAGATGAAATTGAGGCATTTCTGGCACCGGCAGGTGTAAAATAA |
| *RcUGT1* | ATGGACAGCGATAGCCGTCCGCTGCGTGTGTTCTTTTTCCCGTTTATGGCGCACGGTCACCTGATCCCGATGGTGGACATTGCGCGTCTGTTCAGCAGCCAGGGCGTTCACAGCACCATCATTACCACCCCGCTGAACGCGAACTACATCAGCAAAACCACCAGCCTGAGCATTAAGACCCTGCCGTTCCAAGCGAGCAAAGTGGGTCTGCCGGATGGTTGCGAGAACGTTGATATGCTGCCGAGCCCGGACCTGATCTTTAAATTTTTCCAGGCGGCGAACCTGCTGCAAAAGCCGTTCGAAAACCTGCTGGAGCTGGAAAAACCGGACTGCCTGATCAGCGATATCTTCTTCCCGTGGAGCGTGGACAGCGCGGGTAAATTTAACATTCCGCGTCTGGTTTTCCACGGCACCAGCTTTTTCGCGATGTGCGCGATGGAGAGCCTGAAGACCCACAAACCGTATAAGAGCGTGAGCACCGACAGCGAGCCGTTTGTTATCCCGAACCTGCCGGATGAAATTAAAATGACCAAGAGCCAGTTCACCGTGGACGCGTGGGAGGATACCGAAAAAGGTGTTGGTAAGCTGCTGGCGGATGCGCGTGCGAGCGGTCTGCGTAGCTTTGGCGTGGTTGTGAACAGCTTCTACGAGCTGGAACCGGCGTATGCGGACTACTATAAAAACGTGCTGAACATGAAGGCGTGGTGCGTTGGTCCGGTTAGCGTGTGCTACCGTAACGATGAGGAAAAGATCGCGCGTGGCAAGAAAAGCGCGATTGACGATCACGAGTGCCTGAAATGGCTGGAAGGCAAGCAGCCGGACAGCGTTGTGTATGTGTGCTTTGGTAGCGGTGCGAGCTTCCCGGATGAGCAACTGCGTGATATCGCGCTGGGTCTGGAAGATAGCGGCGTGAACTTTATCTGGGTTATTCGTCGTAGCAGCGAGAGCGGTAGCGAAGACTACCTGCCGGAGGGCTTCGAAGATCGTGTGGAAGGTAGCGGTCTGGTTATCCGTGGCTGGGCGCCGCAAGTTCTGATTCTGGACCACCCGAGCGTGGGTGGCTTTGTTACCCACTGCGGTTGGAACAGCGCGCTGGAGGGTATTAGCGCGGGTCTGCCGATGGTGACCTGGCCGCTGTTCGCGGAACAGTTTTTCAACCAAAAACTGATCACCGACGTTCTGAAGATTGGTGTTGAGGTGGGCGTTCAGAAGTGGAGCCGTAACGGCGAAGATCGTGTGACCAAGGAGAAAGTGGAAAAAGCGGTTCGTGCGGTGATGGTTGGTGAGGTTGCGGAGGAACGTCGTGGTCGTGCGCGTCAACTGGGCAAACTGGCGAAGAACGCTGCGGCGAAGGATGGTAGCAGCTTCATCGACCTGCACAACCTGCTGGATGAACTGAAACTGCGTCGTGTTCCGCTGAGCTAA |
| *RrUGT3* | ATGTCTGGCACCCCACACATCGCCATCCTGCCGAGCCCGGGCATGGGCCACCTGATCCCGATGGCCGAGTTCGCCAAGCGCCTGGTTCACCACCACAACTTCTCTATCACCTTCGTTATCCCGACCGACGGCCCACCGTCCTCCGCCTACCAACAAGTTCTGACCTCCCTGCCATCTTCCATCGATCACATCTTCCTGCCACAAGTTGACCTGACCGACGTTGTATCTCAATCTCCAGCTCATCCGCGTATCGAAACCCTGATCTCCCTGACCGTTGCTCGCTCCCTGTCCTCCCTGCGCACCACCCTGTCCTCTCTGCAATCTTCTAAAAACCTGGTTTCTCTGGTTGTTGATCTGTTCGGCACTGATGCATTCGACCCGGCCATCGAGCTGGGCATCTCTCCGTACATTTTCTTCCCGTCCACCGCCATGACCCTGTCTCTGTTCCTGTACATGCCGCAGCTGGACAAATCTGTTACCTGCGAATTTCGTCACATGACCGATCTGGTTCGTATTCCGGGTTGCGTTCCGGTTCGTGGTTCTGATCTGTTCGACCCGGTTCAAGACCGTACCGACGAGGCTTATAAATGGGTTATCCATCACTCCAACCGTTACCCGATGGCGGAGGGTGTTATCGAGAACAGCTTCATGGAGCTGGAACATGGTGCGCTGAAGTATCTGCAAACCGTTCAATCTGGTAAGCCGCCGGTTTACGCGGTTGGTCCGCTGATTAAAATGGATTATGATGTTGACGATTCCGGTTCTAAGATCATCGAGTGGCTGGATGATCAACCGGTTGGTTCTGTTCTGTTTGTTTCTTTTGGTAGCGGCGGTACTCTGTCTTATGAGCAAATGACCGAGCTGGCTCACGGTCTGGAATCTAGCCAGCAACGTTTCCTGTGGGTGGTTCGTTCTCCGAACCAAATCCCGAACAGCACCTATTTCTCTGTACAAAGCCAAAAAGACCCGCTGGCTTACCTCCCAGAAGGCTTCCTGAACCGTACCGAGGGTCGTGGTCTGGTTGTATCTAACTGGGCCCCACAGGCTCAAATTCTGTCTCACGGCTCTACTGGTGGCTTCATGAGCCACTGCGGTTGGAACTCTATTCTGGAGTCTGTGGTGCACGGCGTGCCGATCATCGCGTGGCCGCTGTACGCCGAGCAGAAGATGAACTCTATCATCGTGGTGGAGGACGTTAAGGTGGCGCTGCGTCCGGCGGGTGTAGGTGAGCGTGTGGTGGAGCGTTCTGAGATCACCGCAGTGGTGAAGGCGCTGATGGAGGGTGAGGAGGGTAAGAAGGTACGTAACCGTATGAAGGAACTGAAGGAAGCGGCGGCACGTGCGGTTTCTGATGACGGTGCGTCTACCATCGCGATTGCGGACCTGGCGCAAAAATGGCGTTCTTCTATGAAGCATTAA |
| *RsUGT72B14* | ATGGCTGGTTCTGGTACTGGTGCTCCGCACATCGCTCTGCTGCCGTCTCCGGGTATGGGTCACCTGATCCCGATGGCTGAATTTGCTAAACGTCTGGTTCACCACCACAACTTCACCGTTACCTTCATCATCCCGACCGACGGTCCGCCGTCTGCTGCTTACCGTCAGGTTCTGGCTTCTCTGCCGACCTCTATCTCTCACATCTTCCTGCCGCCGGTTGACCTGTCTGACGTTGTTCCGTCTCACCCGCGTATCGAAACCCTGATCTCTCTGACCGTTGTTCGTTCTCTGCCGTCTCTGCACAACACCATCGCTTCTCTGCTGGCTTCTAAAAACCTGGCTGCTCTGTTCGTTGACCTGTTCGGTACTGACGCTTTCGACCCGGCTATCGACCTGGGTGTTTCTCCGTACATCTTCTTCCCGTCTACCGCTATGACCCTGTCTCTGATCCTGCACATGCCGGAACTGGACCGTTCTGTTACCTGCGAATACCGTCACATGACCGACCTGGTTCGTATCCCGGGTTGCATCCCGATCCGTGGTTCTGACCTGTTCGACCCGGTTCAGGACCGTACCGACGAAGCGTACAAACGTATCGTTCACCACGCTAAACGTTACCCGATGGCTGAAGGTATCATCGAAAACTCTTTCATGGAACTGGAACCGGGTGCTCTGAAATACCTGCAATCTGTTGAACCGGGTCGTCCGCCGGTTTACGCTGTTCGTCCGCTGATCAAAATGGACTACGAAGTTGACTCTTCTGGTTCTAAAATCATCGAATGGCTGGACGGTCAGCCGATCGGTTCTGTTCTGTTCATCTCTTTCGGTTCTGGTGGTACTCTGTCTTTCGACCAGATGACCGAACTGGCTCACGGTCTGGAATCTTCTCAGCAGCGTTTCCTGTGGGTTGTTCGTTCTCCGTCTCTGATCCCGAACTCTGCTTACTTCTCTGCTCAGTCTCAGAACGACCCGCTGGCTTACCTGCCGGACGGTTTCCTGAACCGTACCTCTGACCGTGGTCTGGTTGTTCCGAACTGGGCTCCGCAGGCTCAGATCCTGTCTCACGGTTCTACCGGTGGTTTCATGTCTCACTGCGGTTGGAACTCTATCCTGGAATCTGTTGTTTACGGTGTTCCGATCATCGCTTGGCCGCTGTACGCTGAACAGAAAACCAACTCTATCATCGTTGTTGAAGACGTTAAAGTTGCTGTTCGTCCGGCTGGTGTTGGTGAAGGTCTGGTTAAACGTCTGGAAGTTGCTACCGCTGTTAAAGCTCTGATGGAAGGTGAAGAAGGTAAAAAAGTTCGTAACCGTATGCGTGACCTGAAAGACGCTGCTGCTCGTGCTATCTGCGTTGACGGTGCTTCTACCAAAGCTATCGCTGAACTGGCTAAAAAATGGCGTTCTTCTGTTAAACACTAA |
